# Up to What Extent Does Dravet Syndrome Benefit From Neurostimulation Techniques?

**DOI:** 10.3389/fneur.2022.843975

**Published:** 2022-04-13

**Authors:** Jiangwei Ding, Lei Wang, Wenchao Li, Yangyang Wang, Shucai Jiang, Lifei Xiao, Changliang Zhu, Xiaoyan Hao, Jiali Zhao, Xuerui Kong, Ziqin Wang, Guangyuan Lu, Feng Wang, Tao Sun

**Affiliations:** ^1^Ningxia Key Laboratory of Cerebrocranial Disease, The Incubation Base of National Key Laboratory, Ningxia Medical University, Yinchuan, China; ^2^Department of Neurosurgery, General Hospital of Ningxia Medical University, Yinchuan, China; ^3^Department of Neurosurgery, The First Affiliated Hospital of Xinxiang Medical University, Weihui, China; ^4^Department of Neurology, First Affiliated Hospital of Zhengzhou University, Academy of Medical Sciences of Zhengzhou University, Zhengzhou, China; ^5^Department of Neurosurgery, The First Affiliated Hospital, Zhejiang University School of Medicine, Hangzhou, China

**Keywords:** Dravet syndrome, drug-resistant epilepsy, neuromodulation, vagus nerve stimulation, deep brain stimulation, transcranial magnetic stimulation

## Abstract

**Background:**

Dravet syndrome (DS) is a refractory developmental and epileptic encephalopathy (EE) with a variety of comorbidities, including cognitive impairment, autism-like behavior, speech dysfunction, and ataxia, which can seriously affect the quality of life of patients and impose a great burden on society and their families. Currently, the pharmacological therapy is patient dependent and may work or not. Neuromodulation techniques, including vagus nerve stimulation (VNS), deep brain stimulation (DBS), transcranial magnetic stimulation (TMS), responsive neurostimulation (RNS), and chronic subthreshold cortical stimulation (CSCS), have become common adjuvant therapies for neurological diseases, but their efficacy in the treatment of DS is unknown.

**Methods:**

We searched Web of Science, PubMed, and SpringerLink for all published cases related to the neuromodulation techniques of DS until January 15, 2022. The systematic review was supplemented with relevant articles from the references. The results reported by each study were summarized narratively.

**Results:**

The Web of science, PubMed and SpringerLink search yielded 258 items. A total of 16 studies published between 2016 and 2021 met the final inclusion criteria. Overall, 16 articles (109 cases) were included in this study, among which fifteen (107 patients) were involved VNS, and one (2 patients) was involved DBS. After VNS implantation, seizures were reduced to ≥50% in 60 cases (56%), seizure free were found in 8 cases (7.5%). Only two DS patients received DBS treatment, and the initial outcomes of DBS implantation were unsatisfactory. The seizures significantly improved over time for both DBS patients after the addition of antiepileptic drugs.

**Conclusion:**

More than half of the DS patients benefited from VNS, and VNS may be effective in the treatment of DS. However, it is important to note that VNS does not guarantee improvement of seizures, and there is a risk of infection and subsequent device failure. Although DBS is a safe and effective strategy for the treatment of refractory epilepsy, the role of DBS in DS needs further study, as the sample size was small. Thus far, there is no strong evidence for the role of DBS in DS.

## Introduction

Epileptic encephalopathy (EE) generally refers to severe cognitive and behavioral impairments resulting from epileptic activity. Such impairments can worsen over time, and the extent of these impairments often exceeds what would be expected from the underlying pathology alone (1). Dravet syndrome (DS), also known as severe infantile myoclonic epilepsy, is a severe EE primarily caused by haploinsufficiency of the *SCN1A* gene, which encodes the brain voltage-gated sodium channel Na_V_1.1 (2, 3). Similarly, gene missense or point mutation mutations in *SCN2A, SCN8A, SCN1B, PCDH19, GABRA1, GABRG2, STXBP1, HCN1, CHD2*, and *KCNA2* can also cause DS or DS-like symptoms (4).

**Figure 1:**
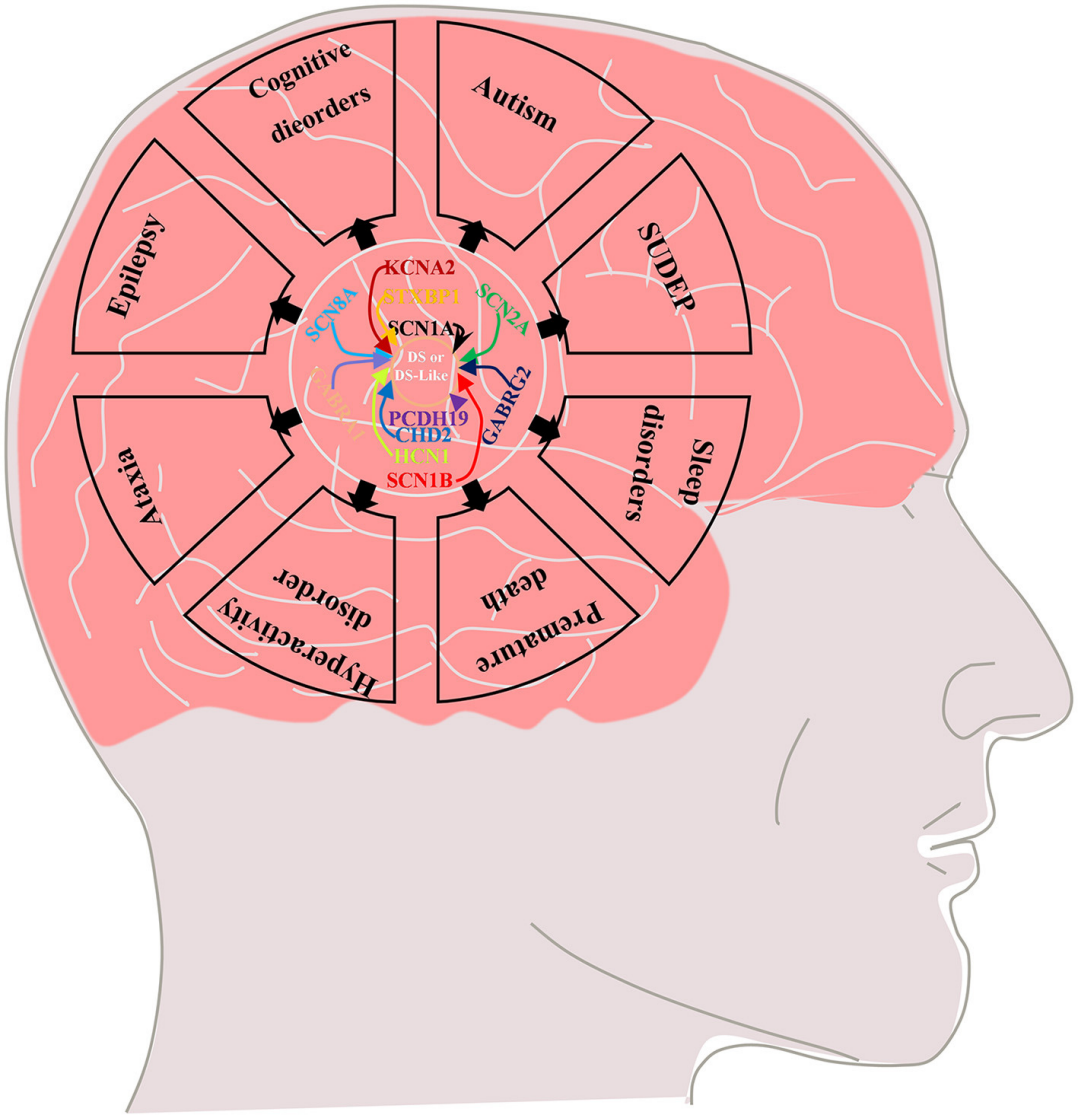
Multiple genetic mutations cause DS or DS-like phenotypes and lead to epilepsy and its comorbidities including cognitive dysfunction (motor, language, and intellectual deficits), autistic behavior, ataxia, sleep disorders, SUDEP and premature death.

Febrile seizure is a typical feature of the early stage (“febrile” phase) of DS. Patients usually have seizures (mostly clonic generalized and unilateral motor seizures) after a fever, vaccination, or warm bath in the first year of life (usually between 4 and 8 months) and often progress to status epilepticus (5–7). This phase is followed by the “worsening” phase at the age of 1–4 years, characterized by the presence of additional seizure types (such as generalized motor, atypical, myoclonic, and absence seizures) with cognitive, behavioral, and motor impairments in which thermogenic factors can still induce seizures (5–8). Finally, the “worsening” phase is followed by the “stabilization” phase, in which the frequency of seizures is reduced compared with the febrile stage (generalized tonic–clonic seizures and tonic seizures are still observed), but cognitive and psychomotor disorders and ataxia are significantly aggravated in the previous phase (5, 7–9). In addition to difficult-to-control epilepsy, DS is often associated with some serious comorbidities, including cognitive impairment, premature death, autism, sleep disorders, hyperactivity, ataxia, and sudden unexpected death in epilepsy (SUDEP) (Figure 1) (9–12), which seriously affect the quality of life of affected children and impose heavy burdens on society and family.

**Figure 2:**
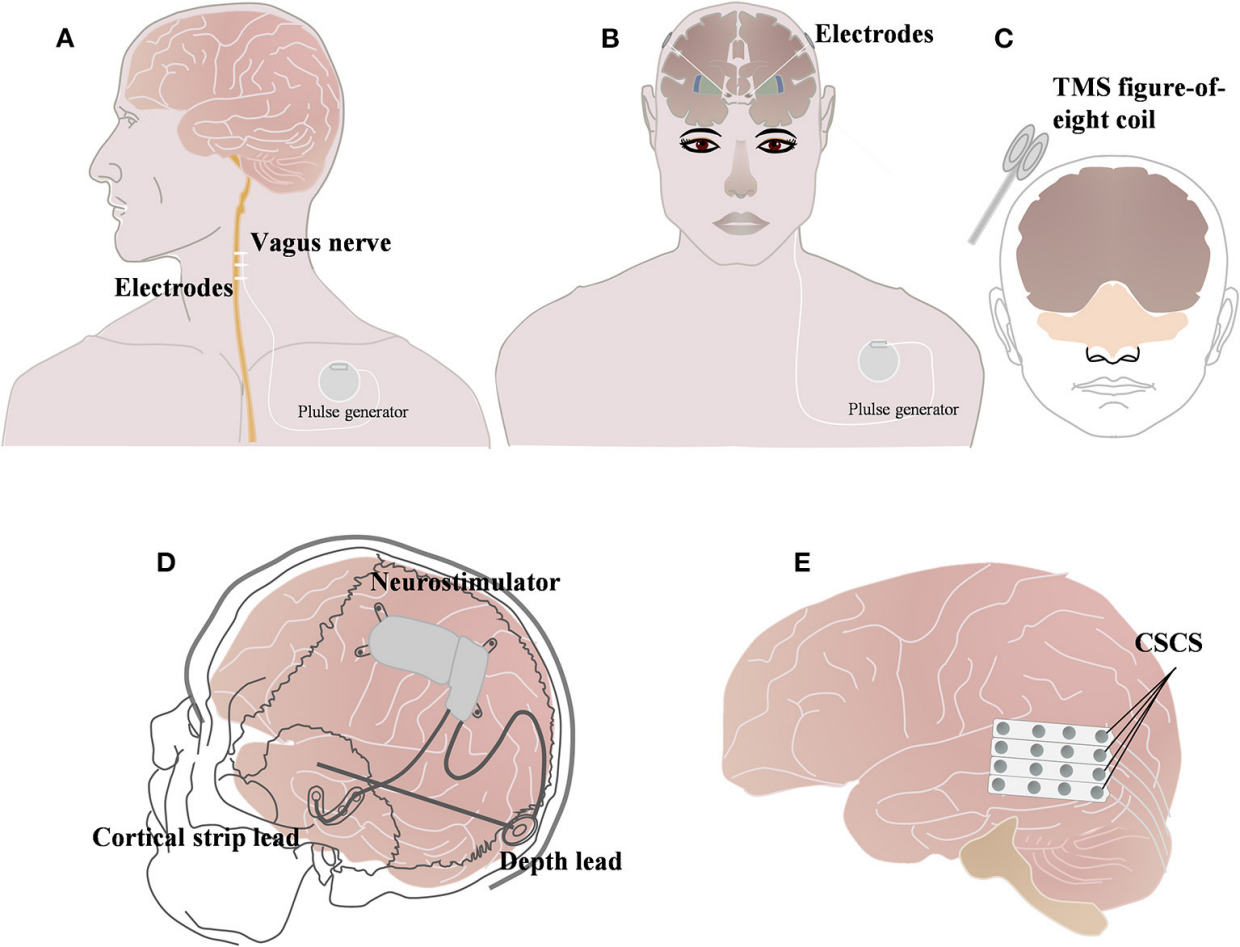
Brief schematic diagram of three neuromodulation techniques commonly used in Dravet syndrome. **(A)** Vagus nerve stimulation (VNS); **(B)** Deep brain stimulation (DBS); **(C)** Transcranial magnetic stimulation (TMS). **(A)** Vagus nerve technique (VNS); **(B)** Deep brain stimulation (DBS); **(C)** Repetitive transcranial magnetic stimulation (rTMS) (13); **(D)** Responsive neurostimulation (RNS) (18); **(E)** and chronic subthreshold cortical stimulation (CSCS) (20).

Neuromodulation, including vagus nerve stimulation (VNS), deep brain stimulation (DBS), and transcranial magnetic stimulation (TMS), responsive neurostimulation (RNS), and chronic subthreshold cortical stimulation (CSCS) (Figure 2), has been widely used in drug-resistant epilepsy (DRE), drug-resistant depression, Parkinson's disease, and other neurologic diseases (Figure 2) (13–19), VNS is the most commonly used of these neuromodulation techniques. Currently, approximately 1,00,000 patients worldwide have received VNS implants (20), but the effectiveness of neuromodulation in DS has rarely been evaluated.

## Methods

### Literature Search

**Figure 3:**
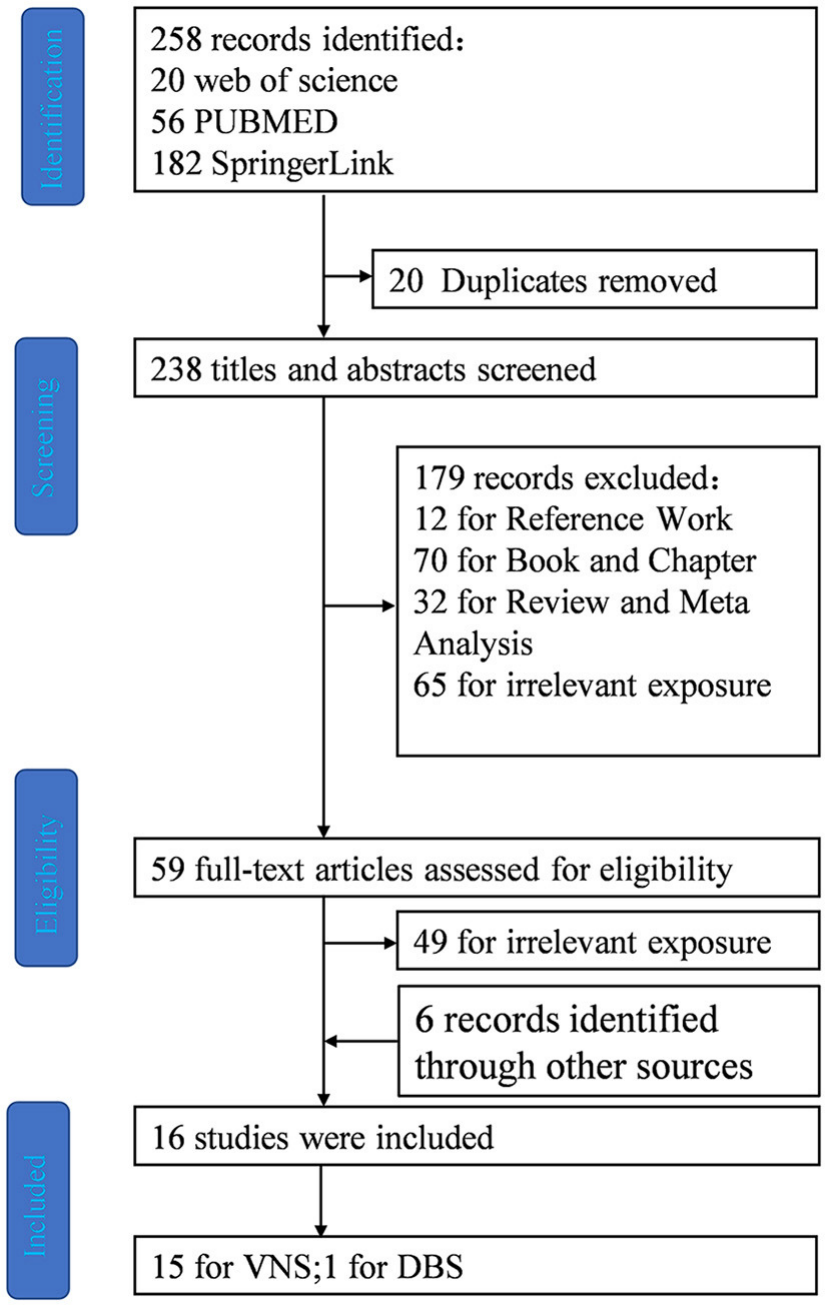
Flow diagram depicting search process and study selection.

A systematic search was performed in Web of science, PubMed and SpringerLink. The most recent search was performed on January 15, 2022, using the term (Dravet Syndrome) AND [(VNS) OR (DBS) OR (TMS) OR (RNS) OR (CSCS)]. We also screened references from the published review papers on VNS and Dravet syndrome. References from relevant articles were used to supplement the systematic review (Figure 3).

### Data Extraction

We excluded articles not written in English or Chinese, if any. Non-original work that has nothing to do with people, such as reviews, meta-analysis, animals or cells, experimental articles not adding information to the question posed in this review, and papers that could not be retrieved via PubMed or SCOPUS. The records were screened by JD and evaluated by LW with respect to the inclusion and exclusion criteria. Disagreements were resolved through a discussion between the two review authors.

### Study Selection Criteria

Inclusion criteria: (i) all neuromodulation techniques related to DS, (ii) effective data between DS and neuromodulation techniques that can be extracted independently, and (iii) not only must the neuromodulation techniques be applied, but also the purpose of epilepsy improvement in DS.

Exclusion criteria: (i) non-neuromodulation techniques, such as antiepileptic drugs, and resection; (ii) DS mixed with other refractory epilepsy disease so that data cannot be summarized for the treatment effect of DS; (iii) only neuromodulation techniques applied but no focus on therapeutic effects for epilepsy; and (iv) unpublished studies, case reports, comments, practice guidelines, reviews, or letters.

## Results

After the elimination of duplicates (20 articles), the literature search yielded 238 articles (Figure 1). After screening all the abstracts, 179 records were excluded. Thus, 59 articles were included in the full-text analysis. Of these, 49 full-text articles were excluded. Six records were identified and supplemented by references to other articles.

Finally, 16 studies met the inclusion and did not meet the exclusion criteria (Figure 1).

### Summary of Findings

According to the previous description, in this study, we still defined the improvement of epilepsy control (responder) as a reduction of more than 50% in generalized tonic-clonic or secondarily generalized tonic-clonic seizures. Patients were followed up for at least 6 months after implantation, otherwise, there was no improvement (responder) (21).

Sixteen articles were eligible in the study, among which 15 (107 patients) were involved in the treatment of DS by VNS (21–35), and one (2 patients) was involved in the treatment of DS by DBS (36) and a total of 107 patients with DS were implanted with VNS, of which 60 (56%) had good epileptic control reduced to more than 50%, and the remaining 47(44%) patients had unsatisfactory epileptic control (Table 1). Eight of the patients were seizure-free, and although most of the adverse effects of VNS implantation were not reported, hoarser was the major side effect and weight loss was reported in one case. The clinical experience of DBS in DS is limited. Two patients with anterior thalamic nucleus stimulation had good epileptic control over time, and their seizures were reduced by more than 90% (Table 2).

**Table 1:** Clinical data of DS patients with VNS implantation.

**References**	**DS case**	**AVI**	**Follow up**	**Responders**	**Non-responders**	**Other interventions**	**Seizure response**	**Adverse events**
Youn et al. (32)	22	10.0 y	4.3 y	12	10	ASMs	36.4 % (8/22), 54.5 % (12/22), and 63.2 % (12/19) had ≥50% seizure reduction at 12, 24, and 36 months, respectively, and 13.3% (3/22) had seizure free ≥1y	Hoarseness (4/22, 18.2 %)
Wang et al. (37)	20	11.8 (6–19) y	2 y	10	10	ASMs	50% (10/20) ≥50% seizure reduction at 24 months	NR
Fulton et al. (21)	20	6.7 (1.9–16) y	2–10 y	13	7	NR	65% (13/20) ≥50% seizure reduction, and 25% (5/20) had seizure free at 6 months	NR
Sirsi et al. (29)	8	6.2 y	2–13 y	4	4	ASMs	50% (4/8) ≥50 % seizure reduction	NR
Dlouhy et al. (22)	6	4.3 y	6.6 y	4	2	VNS,CC	67% (4/6) ≥50% seizure reduction*	NR
Fernandez et al. (23)	2	2.2 y, 2.8 y	3 y	2	0	ASMs	100%(2/2) ≥50% seizure reduction at 12 months	NR
Dressler et al. (24)	8	NR	3 m	3	5	ASMs	38% (3/8) ≥50% seizure reduction 3m	NR
Spatola et al. (27)	1	19 y	3 m	1	0	ASMs	>90% seizure reduction	NR
Chen et al. (34)	1	NR	24 m	1	0	ASMs	>90% seizure reduction	Hoarseness
Cersósimo et al. (33)	3	14 (13,14,15)	26 (23, 26, 30) m	2	1	NR	67% (2/3) ≥50% seizure reduction	Hoarseness, coughing
Caraballo et al. (25)	3	NR	NR	2	1	ASMs	67% (2/3) had ≥50% seizure reduction	NR
Zamponi et al. (26)	8	10.3 (5–25)	1 y	4	4	ASMs	50% (4/8) had ≥50% seizure reduction at 12 months	NR
Shahwan, et al. (35)	2	5.7, 11.8	6 and 7.5 m	1	1	ASMs	50% (1/2) ≥50% seizure reduction and SUDEP**#**	Weight loss
Rossignol et al. (28)	2	NR	2 y	1	1	NR	50% (1/2) had>90% seizure reduction	NR
Kang et al. (31)	1	165 m	12 m	0	1	NR	25% seizure reduction	Hoarseness
Total	107	/	/	60 (56%)	47 (44%)	Other interventions	7.5% (8/107) had seizure free and 56%(60/107) had>50% seizure reduction	

*NR, No recorded; AVI, age at VNS implantation.*

*One of the patients who underwent corpus callotomy after VNS implantation had a 50% reduction in seizures and was not counted.

*^#^Although the patient's epilepsy was well controlled after VNS implantation, SUDEP was not avoided (6 months after VNS implantation)*.

**Table 2:** Clinical data of DS patients with DBS implantation.

**Study**	**Case**	**Gender**	**Age of onset**	**ADI**	**Stimulating nuclei**	**Follow up**	**Seizure response**	**Adverse events**
Andrade et al. (36)	1	M	1.5 y	19 y	Anterior nucleus (AN) thalamic	9.5 y	GTCS >90% seizure reduction	NR
	2	F	1 y	34 y	Anterior nucleus (AN) thalamic	10 y	67–93% seizure reduction	NR

*NR, No recorded; ADI, age at DBS implantation*.

## Discussion

DS is a special type of DRE. Despite the emergence of new antiseizure medications (ASMs; such as cannabidiol, CBD; stiripentol, STP; and fenfluramine, FFA) in recent years, the treatment of DS is still challenging (38). Neuromodulation techniques as a minimally invasive or non-invasive approach is a promising treatment for neurologic disorders. Our objective in this review was to demonstrate the efficacy of neuromodulation techniques, especially VNS, in DS and to provide a treatment option for patients with DS.

### Effect of ASMs on Dravet Syndrome

#### Conventional ASMs Therapy

The treatment of DS follows an individualized treatment regimen, but medication is only partially effective for DS seizures. Commonly used sodium channel blockers such as carbamazepine and lamotrigine may exacerbate seizures or even cause epileptic status, and may also cause further deterioration of cognitive function. Control of seizures often requires a combination of antiepileptic drugs (AEDs), of which valproate and clobazam are considered first-line treatments for DS (38–41). Ketogenic diet (KD) have shown promise in the treatment of DS and have been effective in animal models of DS (41, 42).

#### Novel ASMs Therapy

##### Cannabidiol

CBD is one of the most abundant plant-derived cannabinoids. CBD, as a non-psychoactive agent, has pharmacological properties of anti-epilepsy (43–45). The United States Food and Drug Administration (FDA) has approved CBD for two childhood-onset EE: DS and Lennox-Gastaut syndrome (LGS) (46, 47). In 2017,Devinsky et al. conducted a double-blind controlled trial of 120 patients with DS and found that 43% of the patients in the CBD group (oral, 20 mg/kg/day) had at least a 50% reduction in seizures compared with a 27% reduction in the placebo control group (48). Miller et al.'s double-blind evaluation of the efficacy of different doses of CBD for DS showed that the oral administration of 10 and 20 mg/kg/day resulted in seizure control rates of 48.7% and 45.7%, respectively (49). Recently, seizures were reduced to 50 in 71% of patients a long-term open-label extension trial (50). Although CBD has been a great success for patients with DS (45, 51), it still fails in 29% to 57% of patients (48, 50). In addition, in a retrospective analysis, CBD was found to be effective in only 3/17 patients and reduced seizures by only >30% (52). Some objective factors, such as CBD is illegal in some countries including mainland China, which also limits the use of CBD to a certain extent (53). Adverse reactions to CBD include diarrhea, vomiting, fatigue, fever, drowsiness, and abnormal liver function (45, 48) (Table 3).

**Table 3:** Representative studies of novel ASMs for DS.

**AEDs**	**Study**	**Study design**	**Recommended dose**	**Concomitant AEDs**	**Response**	**No-Response**	**Side effect**
CBD	Devinsky et al. (48)	Double-blind, placebo-controlled trial	2–5 mg/kg/d (Initial dose) and 25 mg/kg/d (maximum dose)	Clobazam; valproate, all forms; stiripentol; levetiracetam; topiramate	43% seizure reduction ≥50%	57%	Diarrhea, vomiting, fatigue, fever, drowsiness, abnormal liver function, decreased appetite.
	Miller et al. (49)	An open-label extension trial	10 mg/kg/d(14weeks)	Valproate (all forms);clobazam; stiripentol; levetiracetam; topiramate	48.7% seizure reduction ≥50%	51.3%	
	Devinsky et al. (54)	Double-blind, placebo-controlled trial	2.5 to 20 mg/kg/d (Initial dose) and 30 mg/kg/d (maximum dose) y(48 weeks)	Clobazam; valproic acid; stiripentol; levetiracetam; topiramate	51% seizure reduction ≥50%	49%	
	Scheffer et al. (50)	An open-label extension trial	≤ 20 mg/kg/day, >20–25 mg/kg/day, >25 mg/kg/day(156weeks)	Valproic acid; clobazam; stiripentol; levetiracetam; topiramate	71% seizure reduction ≥50%	29%	
	Madan Cohen et al. (55)	Double-blind, placebo-controlled trial	CBD 10 and 20 mg/ kg/day	Valproate; clobazam; stiripentol; levetiracetam; topiramate	54.1% seizure reduction ≥50%	45.9%	
STP	Inoue et al. (56)	An open-label multicenter study	15–20 mg/kg/d(Initial), 50 mg/kg/d(target) and 100 mg/kg/d(maximum)	Clobazam; valproate bromide; phenobarbital; zonisamide; clonazepam; ethosuximide; phe nytoin; carbamazepine; diazepam	61% (GTCS) had ≥50%	49%	loss of appetite, sleep disturbance, ataxia, and hyperactivity/irritability, fatigue, diarrhea, and pyrexia
FFA	Specchio et al. (58)	A Randomized Clinical Trial	0.2 mg/kg/d(Initial), 0.7 mg/kg/d(maximum)	Clobazam; clonazepam; ethosuximide; levetiracetam; phenobarbital; stiripentol; topiramate; valproic acid; zonisamide	71.1% had a ≥ 50% seizure reduction	28.9%	No echocardiographic signs of cardiac valvulopathy or pulmonary hypertension were observed
	Nabbout et al. (57)	A Randomized Clinical Trial	0.4 mg/kg/d,17 mg/kg/d(maximum)	Stiripentol; clobazam; valproate; topiramate; levetiracetam	54% had≥50% seizure reduction	46%	

*CBD, Cannabidiol; STP, Stiripentol; FFA, Fenfluramine*.

##### Stiripentol

STP is a novel antiepileptic drug with oral activity and unique structure (59, 60). In the European Union and Canada, STP is approved for use in combination with clobacan and valproate as an adjunct treatment for refractory generalized tonic-clonic seizures in patients with DS (infancy). In Japan, STP is approved in combination with clobazam and valproate for the treatment of clonic or tonic-clonic seizures in DS patients with poor response to clobazam and valproate. The United States approved indication for STP is for the treatment of DS related seizures in patients 2 years of age and older taking clobazine (61). Unlike the European Union, Canada and Japan, the United States has an age limit on the use of STP for DS patients and does not specify valproic acid as a required combination drug. STP reduces the frequency of epileptic seizures in DS patients. Compared with other antiepileptic drugs, it acts as an allosteric modulator of GABAAR, and may increase the inhibitory effect of GABA on neurotransmission and enhance the effect of BZ. The initial dose of the drug is 15–20 mg/(kg·d) and the target dose is 50 mg/(kg·d) in 2–4 weeks, with the maximum dose of 100 mg/(kg·d) available for children (56). In a recent study, STP was shown to respond to only 54% of patients (57). Adverse effects commonly observed with STP are dose-dependent and include somnolence, fidgety, irritability, low IOP, nausea, vomiting, loss of appetite and weight. There are also reported risks of elevated γ-glutamyltransferase and neutropenia, so routine tests of liver function and blood are also necessary. Since some of these side effects may be associated with an accompanying increase in valproate or clobazam levels, it is recommended to reduce the dose of the latter two drugs at the onset of STP (Table 3).

##### Fenfluramine

Sullivan et al. administered FFA to 232 DS patients (initial dose 0.2 mg/kg/d, 4 weeks later, the dose of fenfluramine can be adjusted according to efficacy and tolerability, with a maximum dose of 0.7 mg/kg/d, a maximum dose of 0.4 mg/kg/d when combined with STP), which has been shown to reduce the frequency of seizures in patients (62). Specchio et al. (58) enrolled 52 patients with DS with a median age of 8.6 years and found that FFA reduced the median incidence of DS seizures by 77.4%. 32 patients (71.1%) had a ≥50% reduction in seizures, and 24 patients (53.3%) had a ≥75% reduction in seizures, among which 5 patients (11.1%) had good control without seizures (58). The most common adverse reactions included fever (21.6%), nasopharyngitis (19.4%) and loss of appetite (15.9%), without valvular disease or pulmonary hypertension (62) (Table 3).

### Surgery and Ketogenic Diet

#### Surgical Operation

Epilepsy lesions removal is the preferred treatment for intractable focal epilepsy, such as focal cortical dysplasia and hippocampal sclerosis (63–65). However, DS is mainly caused by *SCN1A* gene mutation, which belongs to “whole brain” epilepsy (66), resulting in over-excitability of the whole brain region without obvious focal lesions, and does not belong to the surgical indication for focal resection. The corpus callosotomy is a palliative surgical treatment and used as an adjunct treatment for refractory epilepsy. Dlouhy et al. (22). made a retrospective analysis of 7 DS patients, in which 5 patients only received VNS implantation, 1 patient only received corpus callosotomy, and 1 patient only received corpus callosotomy after VNS due to poor epileptic control. However, it is important to note that corpus callosotomy is not currently recommended for the treatment of Dravet syndrome (38–41).

#### Ketogenic Diet

KD is a diet with a high proportion of fat intake, a moderate proportion of protein intake and a low proportion of carbohydrate intake, which is commonly used as an adjutant non-drug therapy for the treatment of epilepsy in children (67, 68). Although the mechanism of KD is not fully understood, it has benefits in anti-epilepsy and in improving cognitive function and behavior. Caraballo et al. (42) found that epilepsy was significantly controlled in 76.9% of DS patients with a continuous KD for more than 1 year, in which 2 patients (15.4%) had seizure free, and 8 patients (61.5%) had a 75–99% decrease in seizures. A study of 60 Chinese patients with DS also found that KD had a good antiepileptic effect, and with the prolongation of KD use time, the benefits of DS patients increased. Most of the patients had KD effect within 2 weeks. At 12 weeks, 58.3% of the patients had >50% seizure reduction. At 24 weeks and 48 weeks, the percentage of DS patients with >50% reduction in seizures increased to 61.1 and 77.3%, respectively. In addition to epilepsy control, cognitive function improved in 22 patients, language progression in 14 patients, and motor function improved in 13 patients (69). A recent meta-analysis also concluded that 63, 60, and 47% of DS patients had a ≥50% reduction in seizures at 3, 6, and 12 months after KD, and the seizure control rates at 6 and 12 months were 78 and 49%, respectively (37). The KD not only effectively controlled seizures, but also improved cognitive, motor and other behaviors. Even in patients with unreduced seizures, the quality of life was improved, and the number of AEDs reduced to one or two on the ketogenic diet (37, 42). Caraballo et al. (42) believed that KD treatment should be considered immediately after three failed AEDs.

### Neurostimulation Techniques

#### VNS

VNS is the most commonly used neuromodulation for DRE. To date, VNS has been implanted in at least 1,00,000 patients worldwide (20, 70). In addition to DRE, VNS has been approved by the FDA for the treatment of refractory depression, migraine (15, 71, 72), and other central nervous system diseases, such as schizophrenia, addiction, Parkinson's disease (73–75), and non-psychiatric diseases such as rheumatoid arthritis, inflammatory bowel disease, and asthma (76–78).

**Figure 4:**
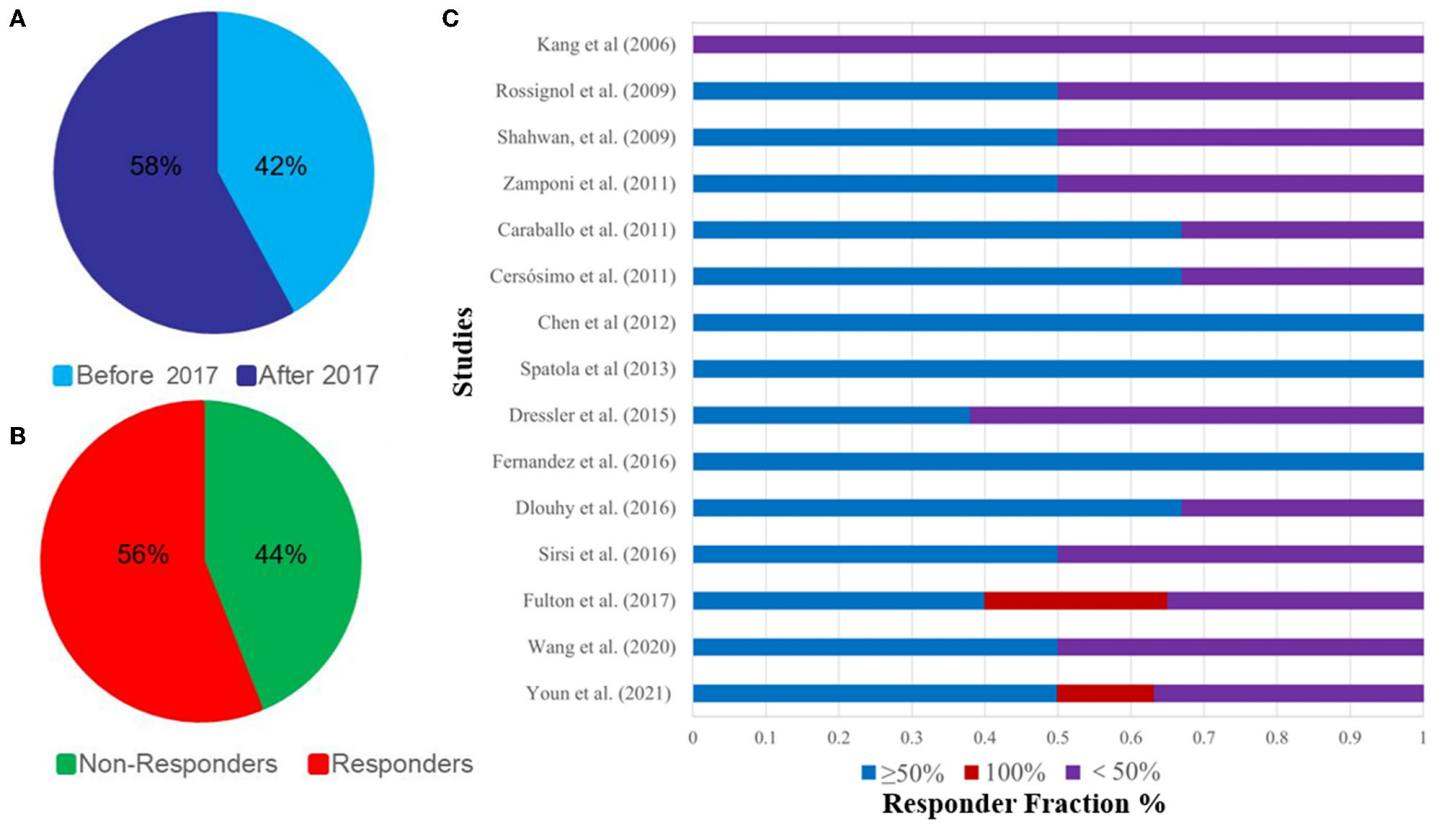
**(A)**, Percentage of DS patients receiving VNS before and after 2017; **(B)**, 15 articles on the epilepsy control rate of VNS in the treatment of DS; **(C)**, Percentage of responders and non-responders to DS receiving VNS.

In 2006, a 165-month-old child with DS received VNS implantation, which may be the first reported case of a child with DS receiving VNS treatment (31). Although seizures were not well controlled in this patient, with only a 25% seizure rate reduction, this has provided new ideas for the treatment of DS. In 2017, the FDA approved VNS for the treatment of DRE in children (79). Since then, an increasing number of DS patients have also received VNS treatment (Table 1). The number of DS patients who received VNS implants after 2017 (62/107, 58%) is significantly more than those receiving them before 2017 (45/107, 42%) (Figure 4A).

The efficacy of VNS for DRE has been widely established. A recent meta-analysis of 101 studies showed that the 50% response rate and seizure freedom were 56.4 and 11.6%, respectively (14). Another study showed that VNS was effective in 54.6% of patients with LGS (80). Obviously, VNS is effective for non-DS refractory epilepsy, but its efficacy against DS, a genetic refractory epilepsy, is our main concern. Dibue-Adjei et al. reported that about 52.9% of patients with VNS had a >50% reduction in seizure rates (81).

Currently, we have included 15 studies involving 107 patients, of which 60 (56%) saw their seizures reduce by ≥50% and eight (7.5%) became seizure-free, indicating that patients with DS can benefit from VNS (Figures 4B,C, Table 1). These results suggest that VNS is equally effective for both DS and non-DS refractory epilepsy.

Similar findings were reported in a meta-analysis by Dibue-Adjei et al. (81), who reported that 52.9% of patients experienced a 50% reduction of seizures. However, since they included only 68 patients in their study, this is slightly lower than our results (56% reduction of seizures), which may be more reliable than Dibue-Adjei et al.'s (81) results since we systematically included the latest studies (30, 32). Although some studies reported hoarseness, coughing, and weight loss in DS patients treated with VNS (31–35) (Table 1), most of them did not describe side effects, and hence we still cannot draw conclusions on tolerability. But despite this, we think these side effects may be insignificant for good seizure improvement. Currently, only VNS has been included in the third-line treatment of DS, and other surgical options, including callosotomy, are not recommended for DS (53).

#### DBS

DBS, which is commonly used to treat movement disorders such as Parkinson's disease, has also been shown to improve the treatment of refractory epilepsy (82–84).

DBS implantation in patients with DS is currently rare, but in two current patients (36). DBS has been shown to significantly reduce epileptic status and appears to be beneficial. In one patient, after ANT-DBS implantation, the frequency of seizures was reduced to 11 generalized tonic-clonic seizures per month (81% reduction). Nine and a half years after DBS implantation, the patient experienced 0.5–1 secondarily generalized tonic-clonic seizures per month. Another DS patient underwent callosotomy at the age of 19 and received ANT-DBS at the age of 36. Levetiracetam and lamotrigine therapy were added in the third and 8 years after the operation, but the seizure frequency changed only slightly. Ten years after DBS implantation, the patient's seizure frequency decreased from 15 seizures per month before DBS to 1–5 seizures per month (Table 2).

## Conclusion

Neuromodulation techniques are a common adjuvant therapy for neurologic diseases. DS is a rare and catastrophic EE. VNS appears to have a positive effect on DS. DBS has been shown to be effective in DRE, but its role in DS is unclear; therefore, a large number of samples and high-quality controlled studies are required.

## Author Contributions

All authors listed have made a substantial, direct, and intellectual contribution to the work and approved it for publication.

## Funding

This study was supported by the National Natural Science Foundation of China, Grant/Award Number: 81971085 and the Advantages Discipline Group Project of Ningxia Medical University, Grant/Award Number: XY201511.

## Conflict of Interest

The authors declare that the research was conducted in the absence of any commercial or financial relationships that could be construed as a potential conflict of interest.

## Publisher's Note

All claims expressed in this article are solely those of the authors and do not necessarily represent those of their affiliated organizations, or those of the publisher, the editors and the reviewers. Any product that may be evaluated in this article, or claim that may be made by its manufacturer, is not guaranteed or endorsed by the publisher.

## References

[1] Scheffer IE Berkovic S Capovilla G Connolly MB French J Guilhoto L. ILAE classification of the epilepsies: position paper of the ilae commission for classification and terminology. Epilepsia. (2017) 58:512–21. 10.1111/epi.13709 28276062 PMC5386840

[2] Marini C Scheffer I Nabbout R Suls A De Jonghe P Zara F. The genetics of Dravet syndrome. Epilepsia. (2011) 52(Suppl. 2):24–9. 10.1111/j.1528-1167.2011.02997.x 21463275

[3] Depienne C Trouillard O Saint-Martin C Gourfinkel-An I Bouteiller D Carpentier W. Spectrum of SCN1A gene mutations associated with Dravet syndrome: analysis of 333 patients. J Med Genet. (2009) 46:183–91. 10.1136/jmg.2008.062323 18930999

[4] Steel D Symonds J Zuberi S Brunklaus AJE. Dravet syndrome and its mimics: beyond SCN1A. Epilepsia. (2017) 58:1807–16. 10.1111/epi.13889 28880996

[5] Miziak B Czuczwar S. Advances in the design and discovery of novel small molecule drugs for the treatment of Dravet Syndrome. Expert Opin Drug Discov. (2021) 16:579–93. 33275464 10.1080/17460441.2021.1857722

[6] Bishop KI Isquith PK Gioia GA Gammaitoni AR Farfel G Galer BS. Improved everyday executive functioning following profound reduction in seizure frequency with fenfluramine: analysis from a phase 3 long-term extension study in children/young adults with Dravet syndrome. Epilepsy Behav. (2021) 121:108024. 10.1016/j.yebeh.2021.108024 34023810

[7] Connolly MB. Dravet syndrome: diagnosis and long-term course. Can J Neurol Sci. (2016) 43(Suppl. 3):S3–8. 10.1017/cjn.2016.243 27264139

[8] Dravet C Oguni H. Dravet syndrome (severe myoclonic epilepsy in infancy). Handb Clin Neurol. (2013) 111:627–33. 10.1016/B978-0-444-52891-9.00065-8 23622210

[9] Brown A Arpone M Schneider AL Micallef S Anderson VA Scheffer IE. Cognitive, behavioral, and social functioning in children and adults with Dravet syndrome. Epilepsy Behav. (2020) 112:107319. 10.1016/j.yebeh.2020.107319 32858363

[10] Kalume F Westenbroek R Cheah C Yu F Oakley J Scheuer T. Sudden unexpected death in a mouse model of Dravet syndrome. J Clin Invest. (2013) 123:1798–808. 10.1172/JCI66220 23524966 PMC3613924

[11] Jansson J Hallböök T Reilly C. Intellectual functioning and behavior in Dravet syndrome: a systematic review. Epilepsy Behav. (2020) 108:107079. 10.1016/j.yebeh.2020.107079 32334365

[12] Ding J Li X Tian H Wang L Guo B Wang Y. SCN1A mutation-beyond Dravet syndrome: a systematic review and narrative synthesis. Front Neurol. (2021) 12:743726. 10.3389/fneur.2021.743726 35002916 PMC8739186

[13] Ammann C Dileone M Pagge C Catanzaro V Mata-Marín D Hernández-Fernández F. Cortical disinhibition in Parkinson's disease. Brain. (2020) 143:3408–21. 10.1093/brain/awaa274 33141146

[14] Jain P Arya RJN. Vagus nerve stimulation and seizure outcomes in pediatric refractory epilepsy: systematic review and meta-analysis. Neurology. (2021). 10.1212/WNL.0000000000012030. [Epub ahead of print]. 33849993

[15] Carreno F Frazer A. Vagal nerve stimulation for treatment-resistant depression. Neurotherapeutics. (2017) 14:716–27. 10.1007/s13311-017-0537-8 28585221 PMC5509631

[16] Torres Diaz C González-Escamilla G Ciolac D Navas García M Pulido Rivas P Sola R. Network substrates of centromedian nucleus deep brain stimulation in generalized pharmacoresistant epilepsy. Neurotherapeutics. (2021) 18:1665–77. 10.1007/s13311-021-01057-y 33904113 PMC8608991

[17] Thuberg D Buentjen L Holtkamp M Voges J Heinze H Lee H. Deep brain stimulation for refractory focal epilepsy: unraveling the insertional effect up to five months without stimulation. Neuromodulation. (2021) 24:373–9. 10.1111/ner.13349 33577139

[18] Skarpaas TL Jarosiewicz B Morrell MJ. Brain-responsive neurostimulation for epilepsy (RNS^®^ System). Epilepsy Res. (2019) 153:68–70. 10.1016/j.eplepsyres.2019.02.003 30850259

[19] Lundstrom BN Worrell GA Stead M Van Gompel JJ. Chronic subthreshold cortical stimulation: a therapeutic and potentially restorative therapy for focal epilepsy. Expert Rev Neurother. (2017) 17:661–6. 10.1080/14737175.2017.1331129 28532252

[20] Toffa D Touma L El Meskine T Bouthillier A Nguyen DJS. Learnings from 30 years of reported efficacy and safety of vagus nerve stimulation (VNS) for epilepsy treatment: a critical review. Seizure. (2020) 83:104–23. 10.1016/j.seizure.2020.09.027 33120323

[21] Fulton S Van Poppel K McGregor A Mudigoudar B Wheless J. Vagus nerve stimulation in intractable epilepsy associated with SCN1A gene abnormalities. J Child Neurol. (2017) 32:494–8. 10.1177/0883073816687221 28079431

[22] Dlouhy B Miller B Jeong A Bertrand M Limbrick D Smyth M. Palliative epilepsy surgery in Dravet syndrome-case series and review of the literature. Childs Nerv Syst. (2016) 32:1703–8. 10.1007/s00381-016-3201-4 27465677

[23] Fernandez L Gedela S Tamber M Sogawa Y. Vagus nerve stimulation in children less than 3 years with medically intractable epilepsy. Epilepsy Res. (2015) 112:37–42. 10.1016/j.eplepsyres.2015.02.009 25847337

[24] Dressler A Trimmel-Schwahofer P Reithofer E Mühlebner A Gröppel G Reiter-Fink E. Efficacy and tolerability of the ketogenic diet in Dravet syndrome - Comparison with various standard antiepileptic drug regimen. Epilepsy Res. (2015) 109:81–9. 10.1016/j.eplepsyres.2014.10.014 25524846

[25] Caraballo R. Nonpharmacologic treatments of Dravet syndrome: focus on the ketogenic diet. Epilepsia. (2011) 52(Suppl. 2):79–82. 10.1111/j.1528-1167.2011.03009.x 21463287

[26] Zamponi N Passamonti C Cappanera S Petrelli C. Clinical course of young patients with Dravet syndrome after vagal nerve stimulation. Eur J Paediatr Neurol. (2011) 15:8–14. 10.1016/j.ejpn.2010.09.003 20971664

[27] Spatola M Jeannet P Pollo C Wider C Labrum R Rossetti A. Effect of vagus nerve stimulation in an adult patient with Dravet syndrome: contribution to sudden unexpected death in epilepsy risk reduction? Eur Neurol. (2013) 69:119–21. 10.1159/000345132 23207687

[28] Rossignol E Lortie A Thomas T Bouthiller A Scavarda D Mercier C. Vagus nerve stimulation in pediatric epileptic syndromes. Seizure. (2009) 18:34–7. 10.1016/j.seizure.2008.06.010 18657451

[29] Sirsi D Khan M Arnold S. Vagal nerve stimulation: is it effective in children with Dravet syndrome? J Pediatr Epilepsy. (2016) 05:007–10. 10.1055/s-0035-1567851

[30] Wang ZJ Kim ES Noh BH Liang JG Lee D Hur YJ. Alteration in brain connectivity in patients with Dravet syndrome after vagus nerve stimulation (VNS): exploration of its effectiveness using graph theory analysis with electroencephalography. J Neural Eng. (2020) 17:036014. 32380482 10.1088/1741-2552/ab914f

[31] Kang HC Hwang YS Kim DS Kim HD. Vagus nerve stimulation in pediatric intractable epilepsy: a Korean bicentric study. Acta Neurochir Suppl. (2006) 99:93–6. 10.1007/978-3-211-35205-2_18 17370772

[32] Youn SE Jung DE Kang HC Kim HD. Long-term results of vagus nerve stimulation in children with Dravet syndrome: time-dependent, delayed antiepileptic effect. Epilepsy Res. (2021) 174:106665. 10.1016/j.eplepsyres.2021.106665 34000601

[33] Cersósimo RO Bartuluchi M De Los Santos C Bonvehi I Pomata H Caraballo RH. Vagus nerve stimulation: effectiveness and tolerability in patients with epileptic encephalopathies. Childs Nerv Syst. (2011) 27:787–92. 10.1007/s00381-010-1314-8 21038079

[34] Chen CY Lee HT Chen CC Kwan SY Chen SJ Hsieh LP. Short-term results of vagus nerve stimulation in pediatric patients with refractory epilepsy. Pediatr Neonatol. (2012) 53:184–7. 10.1016/j.pedneo.2012.04.005 22770107

[35] Shahwan A Bailey C Maxiner W Harvey AS. Vagus nerve stimulation for refractory epilepsy in children: more to VNS than seizure frequency reduction. Epilepsia. (2009) 50:1220–8. 10.1111/j.1528-1167.2008.01940.x 19170732

[36] Andrade D Hamani C Lozano A Wennberg R. Dravet syndrome and deep brain stimulation: seizure control after 10 years of treatment. Epilepsia. (2010) 51:1314–6. 10.1111/j.1528-1167.2009.02408.x 19919661

[37] Wang Y Fang Z Zhang Y Xie L Jiang L. Efficacy of the ketogenic diet in patients with Dravet syndrome: a meta-analysis. Seizure. (2020) 81:36–42. 10.1016/j.seizure.2020.07.011 32712377

[38] Wirrell EC Nabbout R. Recent advances in the drug treatment of Dravet syndrome. CNS Drugs. (2019) 33:867–81. 10.1007/s40263-019-00666-8 31549357

[39] Nabbout R Copioli C Chipaux M Chemaly N Desguerre I Dulac O. Ketogenic diet also benefits Dravet syndrome patients receiving stiripentol: a prospective pilot study. Epilepsia. (2011) 52:e54–7. 10.1111/j.1528-1167.2011.03107.x 21569025

[40] Cardenal-Muñoz E Auvin S Villanueva V Cross J Zuberi S Lagae L. Guidance on Dravet syndrome from infant to adult care: road map for treatment planning in Europe. Epilepsia Open. (2021). 10.1002/epi4.12569 PMC8886070 34882995

[41] Cross JH Caraballo RH Nabbout R Vigevano F Guerrini R Lagae L. Dravet syndrome: treatment options and management of prolonged seizures. Epilepsia. (2019) 60(Suppl. 3):S39–48. 10.1111/epi.16334 31904119

[42] Caraballo R Cersósimo R Sakr D Cresta A Escobal N Fejerman N. Ketogenic diet in patients with Dravet syndrome. Epilepsia. (2005) 46:1539–44. 10.1111/j.1528-1167.2005.05705.x 16146451

[43] Daniel F Orrin D. Cannabinoids in the treatment of epilepsy. N Engl J Med. (2016) 374:94. 10.1056/NEJMc1512758 26672646

[44] O"Connell BK Gloss D Devinsky O. Cannabinoids in treatment-resistant epilepsy: a review. Epilepsy Behav. (2017) 70:341–8. 10.1016/j.yebeh.2016.11.012 28188044

[45] Villanueva V Carreño-Martínez M Gil Nagel-Rein A López-González FJ. New therapeutic approach in Dravet syndrome and Lennox-Gastaut syndrome with cannabidiol. Rev Neurol. (2021) 72:S1–10. 10.33588/rn.72S01.2021017 33908026

[46] Samanta D. Cannabidiol: A review of clinical efficacy and safety in epilepsy. Pediatr Neurol. (2019) 96:24–9. 10.1016/j.pediatrneurol.2019.03.014 31053391

[47] Devinsky O Patel AD Cross JH Villanueva V Wirrell EC Privitera M. Effect of cannabidiol on drop seizures in the Lennox-Gastaut syndrome. N Engl J Med. (2018) 378:1888–97. 10.1056/NEJMoa1714631 29768152

[48] Devinsky O Cross JH Wright S. Trial of Cannabidiol for Drug-Resistant Seizures in the Dravet Syndrome. N Engl J Med. (2017) 377:699–700. 10.1056/NEJMc1708349 28813226

[49] Miller I Scheffer IE Gunning B Sanchez-Carpintero R Gil-Nagel A Perry MS. dose-ranging effect of adjunctive oral cannabidiol vs. placebo on convulsive seizure frequency in Dravet syndrome: a randomized clinical trial. JAMA Neurol. (2020) 77:613–21. 10.1001/jamaneurol.2020.0073 32119035 PMC7052786

[50] Scheffer IE Halford JJ Miller I Nabbout R Sanchez-Carpintero R Shiloh-Malawsky Y. Add-on cannabidiol in patients with Dravet syndrome: results of a long-term open-label extension trial. Epilepsia. (2021) 62:2505–17. 10.1111/epi.17036 34406656

[51] Ridler C. Epilepsy: Cannabidiol reduces seizure frequency in Dravet syndrome. Nat Rev Neurol. (2017) 13:383. 10.1038/nrneurol.2017.86 28621765

[52] Silvennoinen K Ritter LM Nashef L Hudgell K Balestrini S Sisodiya SM. Two-center experience of cannabidiol use in adults with Dravet syndrome. Seizure. (2021) 91:5–8. 10.1016/j.seizure.2021.05.014 34052628

[53] Wirrell EC Laux L Donner E Jette N Knupp K Meskis MA. Optimizing the diagnosis and management of Dravet syndrome: recommendations from a North American consensus panel. Pediatr neurol. (2017) 68:18–34.e3. 10.1016/j.pediatrneurol.2017.01.025 28284397

[54] Devinsky O Nabbout R Miller I Laux L Zolnowska M Wright S. Long-term cannabidiol treatment in patients with Dravet syndrome: an open-label extension trial. Epilepsia. (2019) 60:294–302. 10.1111/epi.14628 30582156 PMC7379690

[55] Madan Cohen J Checketts D Dunayevich E Gunning B Hyslop A Madhavan D. Time to onset of cannabidiol treatment effects in Dravet syndrome: analysis from two randomized controlled trials. Epilepsia. (2021) 62:2218–27. 10.1111/epi.16974 34265088 PMC8456817

[56] Inoue Y Ohtsuka Y Oguni H Tohyama J Baba H Fukushima K. Stiripentol open study in Japanese patients with Dravet syndrome. Epilepsia. (2009) 50:2362–8. 10.1111/j.1528-1167.2009.02179.x 19552653

[57] Nabbout R Mistry A Zuberi S Villeneuve N Gil-Nagel A Sanchez-Carpintero R. Fenfluramine for treatment-resistant seizures in patients with Dravet syndrome receiving stiripentol-inclusive regimens: a randomized clinical trial. JAMA Neurol. (2020) 77:300–8. 10.1001/jamaneurol.2019.4113 31790543 PMC6902175

[58] Specchio N Pietrafusa N Doccini V Trivisano M Darra F Ragona F. et al. Efficacy and safety of Fenfluramine hydrochloride for the treatment of seizures in Dravet syndrome: a real-world study. Epilepsia. (2020) 61:2405–14. 10.1111/epi.16690 32945537

[59] Plosker G. Stiripentol : in severe myoclonic epilepsy of infancy (Dravet syndrome). CNS Drugs. (2012) 26:993–1001. 10.1007/s40263-012-0004-3 23018548

[60] Nickels K Wirrell E. Stiripentol in the management of epilepsy. CNS Drugs. (2017) 31:405–16. 10.1007/s40263-017-0432-1 28434133

[61] Frampton J. Stiripentol: a review in Dravet syndrome. Drugs. (2019) 79:1785–96. 10.1007/s40265-019-01204-y 31617141

[62] Sullivan J Scheffer I Lagae L Nabbout R Pringsheim M Talwar D. Fenfluramine HCl (Fintepla) provides long-term clinically meaningful reduction in seizure frequency: analysis of an ongoing open-label extension study. Epilepsia. (2020). 10.1111/epi.16722 PMC7756901 33078386

[63] Villanueva V Carreño M Herranz Fernández JL Gil-Nagel A. Surgery and electrical stimulation in epilepsy: selection of candidates and results. Neurologist. (2007) 13(Suppl. 1):S29–37. 10.1097/NRL.0b013e31815c0fbc 18090949

[64] Jehi L. The relation between lesion removal and seizure freedom after epilepsy surgery: all lesions are not created equal. Epilepsy Curr. (2018) 18:170–1. 10.5698/1535-7597.18.3.170 29950941 PMC6017685

[65] Alonso-Vanegas MA Freire Carlier ID San-Juan D Martínez AR Trenado C. Parahippocampectomy as a New Surgical Approach to Mesial Temporal Lobe Epilepsy Caused By Hippocampal Sclerosis: A Pilot Randomized Comparative Clinical Trial. World Neurosurg. (2018) 110:e1063-e71. 10.1016/j.wneu.2017.11.170 29229342

[66] Stein R Kaplan J Li J. Catterall W. Hippocampal deletion of Na11 channels in mice causes thermal seizures and cognitive deficit characteristic of Dravet Syndrome. Proc Natl Acad Sci U S A. (2019) 116:16571–6. 10.1073/pnas.1906833116 31346088 PMC6697805

[67] Murphy P Likhodii S Nylen K Burnham W. The antidepressant properties of the ketogenic diet. Biol Psychiatry. (2004) 56:981–3. 10.1016/j.biopsych.2004.09.019 15601609

[68] Lyons L Schoeler N Langan D Cross J. Use of ketogenic diet therapy in infants with epilepsy: a systematic review and meta-analysis. Epilepsia. (2020) 61:1261–81. 10.1111/epi.16543 32452537

[69] Tian X Chen J Zhang J Yang X Ji T Zhang Y. The efficacy of ketogenic diet in 60 Chinese patients with Dravet syndrome. Front Neurol. (2019) 10:625. 10.3389/fneur.2019.00625 31249551 PMC6584746

[70] Ding J Wang L Wang C Gao C Wang F Sun T. Is vagal-nerve stimulation safe during pregnancy? A mini review Epilepsy Res. (2021) 174:106671. 10.1016/j.eplepsyres.2021.106671 34022523

[71] Yap J Keatch C Lambert E Woods W Stoddart P Kameneva T. Critical review of transcutaneous vagus nerve stimulation: challenges for translation to clinical practice. Front Neurosci. (2020) 14:284. 10.3389/fnins.2020.00284 32410932 PMC7199464

[72] Mauskop A. Vagus nerve stimulation relieves chronic refractory migraine and cluster headaches. Cephalalgia. (2005) 25:82–6. 10.1111/j.1468-2982.2005.00611.x 15658944

[73] Rosso P Iannitelli A Pacitti F Quartini A Fico E Fiore M. Vagus nerve stimulation and neurotrophins: a biological psychiatric perspective. Neurosci Biobehav Rev. (2020) 113:338–53. 10.1016/j.neubiorev.2020.03.034 32278791

[74] García-Toro M Gili M Roca M. New neurostimulation techniques in adicctions. Adicciones. (2011) 23:273–6. 10.20882/adicciones.120 22249892

[75] Farrand A Verner R McGuire R Helke K Hinson V Boger H. Differential effects of vagus nerve stimulation paradigms guide clinical development for Parkinson's disease. Brain Stimul. (2020) 13:1323–32. 10.1016/j.brs.2020.06.078 32629028

[76] Drewes A Brock C Rasmussen S Møller H Brock B Deleuran B. Short-term transcutaneous non-invasive vagus nerve stimulation may reduce disease activity and pro-inflammatory cytokines in rheumatoid arthritis: results of a pilot study. Scand J Rheumatol. (2021) 50:20–7. 10.1080/03009742.2020.1764617 33047630

[77] Bonaz B Picq C Sinniger V Mayol J Clarençon Clarençon D. Vagus nerve stimulation: from epilepsy to the cholinergic anti-inflammatory pathway. Neurogastroenterol Motil. (2013) 25:208–21. 10.1111/nmo.12076 23360102

[78] Miner J Lewis L Mosnaim G Varon J Theodoro D Hoffmann T. Feasibility of percutaneous vagus nerve stimulation for the treatment of acute asthma exacerbations. Acad Emerg Med. (2012) 19:421–9. 10.1111/j.1553-2712.2012.01329.x 22506946

[79] González HFJ Yengo-Kahn A Englot DJ. Vagus nerve stimulation for the treatment of epilepsy. Neurosurg Clin N Am. (2019) 30:219–30. 10.1016/j.nec.2018.12.005 30898273 PMC6432928

[80] Thirunavu V Du R Wu JY Berg AT Lam SK. The role of surgery in the management of Lennox-Gastaut syndrome: a systematic review and meta-analysis of the clinical evidence. Epilepsia. (2021) 62:888–907. 10.1111/epi.16851 33626200

[81] Dibué-Adjei M Fischer I Steiger HJ Kamp MA. Efficacy of adjunctive vagus nerve stimulation in patients with Dravet syndrome: a meta-analysis of 68 patients. Seizure. (2017) 50:147–52. 10.1016/j.seizure.2017.06.007 28666193

[82] Li M Cook M. Deep brain stimulation for drug-resistant epilepsy. Epilepsia. (2018) 59:273–90. 10.1111/epi.13964 29218702

[83] Klinger N Mittal S. Deep brain stimulation for seizure control in drug-resistant epilepsy. Neurosurg Focus. (2018) 45:E4. 10.3171/2018.4.FOCUS1872 30064326

[84] Vitek J Starr P. Studies of deep brain stimulation in Parkinson's disease. Lancet Neurol. (2020) 19:807–8. 10.1016/S1474-4422(20)30323-9 32949537

